# Single Nucleotide Polymorphisms of the *RAC1* Gene as Novel Susceptibility Markers for Neuropathy and Microvascular Complications in Type 2 Diabetes

**DOI:** 10.3390/biomedicines11030981

**Published:** 2023-03-22

**Authors:** Iuliia Azarova, Elena Klyosova, Alexey Polonikov

**Affiliations:** 1 Department of Biological Chemistry, Kursk State Medical University, 3 Karl Marx Street, Kursk 305041, Russia; azzzzar@yandex.ru; 2 Laboratory of Biochemical Genetics and Metabolomics, Research Institute for Genetic and Molecular Epidemiology, Kursk State Medical University, 18 Yamskaya St., Kursk 305041, Russia or klesovaeu@kursksmu.net; 3 Department of Biology, Medical Genetics and Ecology, Kursk State Medical University, 3 Karl Marx Street, Kursk 305041, Russia; 4 Laboratory of Statistical Genetics and Bioinformatics, Research Institute for Genetic and Molecular Epidemiology, Kursk State Medical University, 18 Yamskaya St., Kursk 305041, Russia

**Keywords:** Rac family small GTPase 1 (RAC1), type 2 diabetes mellitus, diabetic neuropathy, diabetic retinopathy, diabetic nephropathy, diabetic angiopathy of the lower extremities, diabetic foot syndrome

## Abstract

Single nucleotide polymorphisms (SNP) in the *RAC1* (Rac family small GTPase 1) gene have recently been linked to type 2 diabetes (T2D) and hyperglycemia due to their contribution to impaired redox homeostasis. The present study was designed to determine whether the common SNPs of the *RAC1* gene are associated with diabetic complications such as neuropathy (DN), retinopathy (DR), nephropathy, angiopathy of the lower extremities (DA), and diabetic foot syndrome. A total of 1470 DNA samples from T2D patients were genotyped for six common SNPs by the MassArray Analyzer-4 system. The genotype rs7784465-T/C of *RAC1* was associated with an increased risk of DR (*p =* 0.016) and DA (*p =* 0.03) in males, as well as with DR in females (*p =* 0.01). Furthermore, the SNP rs836478 showed an association with DR (*p =* 0.005) and DN (*p =* 0.025) in males, whereas the SNP rs10238136 was associated with DA in females (*p =* 0.002). In total, three *RAC1* haplotypes showed significant associations (FDR < 0.05) with T2D complications in a sex-specific manner. The study’s findings demonstrate, for the first time, that the *RAC1* gene’s polymorphisms represent novel and sex-specific markers of neuropathy and microvascular complications in type 2 diabetes, and that the gene could be a new target for the pharmacological inhibition of oxidative stress as a means of preventing diabetic complications.

## 1. Introduction

Over 500 million people worldwide are affected by type 2 diabetes (T2D), which, along with obesity, is the second most frequent endocrinological disease [1]. About 7.8 million people in the Russian Federation have diabetes, with T2D accounting for the majority of cases [1]. Type 2 diabetes is associated with a multitude of disorders in lipid, protein, nucleotide metabolism, and redox homeostasis, leading to long-term complications that made T2D the ninth cause of mortality in 2020 [2]. In particular, impaired redox homeostasis is thought to be an important pathological condition underlying oxidative stress that contributes to the initiation and progression of type 2 diabetes [3,4,5]. Hyperglycemia, an increase in reactive oxygen species (ROS) production with cytosolic NADPH oxidase, and a deficiency in key antioxidants such as reduced glutathione (GSH) are considered to be the major damaging factors that are responsible for the structural and functional alterations in the retina, kidneys, nerves, and vessels in diabetics that ultimately lead to complications such as diabetic retinopathy (DR), diabetic nephropathy (DF), diabetic neuropathy (DN), diabetic angiopathy of the lower extremities (DA), and diabetic foot syndrome (DFS) [6,7,8,9].

The NADPH oxidase (NOX) enzyme is primarily responsible for the generation of superoxide anion radicals, which accumulate excessively in the cell and cause oxidative stress [10]. Once generated, these superoxides are rapidly dismutated into hydrogen peroxide, either spontaneously or via superoxide dismutase [11]. Other ROS are generated through the reactions of the superoxide radical with nitric oxide to form peroxynitrite, the peroxidase-catalyzed formation of hypochlorous acid from hydrogen peroxide, and the iron-catalyzed Fenton’s reaction which produces hydroxyl radicals [12,13]. Notably, increased ROS generation was found to interact with the proteins of the insulin signaling pathway, contributing to insulin resistance [14,15] and triggering the dysfunction and apoptosis of pancreatic beta-cells [16].

The NOX enzyme represents a multi-subunit complex consisting of several proteins, among which small GTPases, such as RAC1 and RAC2, are known to activate the holoenzyme [17]. A total of two experimental studies [18,19] have shown that the transcriptional activation of the *RAC1* gene in diabetic mice has been found to contribute to mitochondrial damage and retinopathy, suggesting at least a causal role of this gene in diabetic complications. We have recently observed that the single nucleotide polymorphisms (SNP) of the *RAC1* gene are tightly associated with impaired redox homeostasis, an increased risk of type 2 diabetes, and hyperglycemia [20]. Pursuing further interests in the role of this gene within T2D, the purpose of the present study was to investigate whether the polymorphisms of the *RAC1* gene contribute to the development of common diabetic complications, such as retinopathy, nephropathy, neuropathy, angiopathy of the lower extremities, and diabetic foot syndrome.

## 2. Materials and Methods

### 2.1. Study Participants and Diagnosis of Type 2 Diabetes

The Regional Ethics Review Committee of Kursk State Medical University gave its approval to the study protocol, which complied with the ethical standards of the Declaration of Helsinki. Before being enrolled in the study, each subject provided their written informed consent. A total of 1470 patients with type 2 diabetes were included in the study. Most of the study participants were Russians from the Kursk region (central Russia). All were patients that had been admitted to the Endocrinology Division of the Kursk Emergency Hospital between November 2016 and October 2019. The following WHO criteria [21,22] were used to verify the T2D diagnosis: a fasting blood glucose (FBG) level of ≥7.0 mmol/L, a random blood glucose level of ≥11.1 mmol/L, and/or a glycated hemoglobin (HbA1c) level of ≥6.5%. The criteria for the inclusion of T2D patients in the study were: (1) a physician-verified diagnosis of T2D, confirmed by clinical, laboratory, and instrumental investigations; (2) an age of over 35 years old; and (3) written informed consent to participate in the study. The criteria for excluding patients from the study were the following: (1) an age of less than 35 years; (2) an absence of written informed consent to participate in the study; and (3) advanced-stage diabetes or the decompensation of diabetes, diabetic coma, immune-mediated or idiopathic type 1 diabetes, gestational diabetes, MODY types of diabetes, diseases of the exocrine pancreas, such as pancreatitis, pancreatic trauma, or pancreatectomy, pancreatic tumors, hereditary diseases affecting the pancreas, or any other endocrine disorders. All of the study’s participants completed a questionnaire [23] on the risk factors of type 2 diabetes.

### 2.2. Genetic Analysis

Fasted venous blood samples were collected from all the study participants, and the genomic DNA was purified by a spin column QIAamp Blood Mini Kit with the use of a robotic workstation QiaCube (QIAGEN, Germany). In total, six commonly tagged SNPs of the *RAC1* gene, such as rs4724800, rs7784465, rs10951982, rs10238136, rs836478, and rs9374, were selected for the study, as described previously [20]. The SNP genotyping was performed using MALDI-TOF mass spectrometry with the MassArray-4 System (Agena Bioscience Inc., San Diego, CA, USA). The primer sequences that were used for the genotyping are available upon request. The genotyping analysis was performed blindly, with regard to the case–control status to ensure quality control. Repeat genotyping was performed on approximately 10% of the samples, randomly selected from the T2D group, and the repeatability test yielded a 100% concordance rate.

### 2.3. Biochemical Analysis

All the biochemical investigations were performed using fasted whole blood samples that were collected in standard sterile tubes with lithium heparin, and immediately centrifuged at 3500 rpm, according to the manufacturer’s instructions (Cell Biolabs, San Diego, CA, USA; Abcam, Waltham, MA, USA). The plasma samples were aliquoted and stored at −80 °C until their further use. For the determination of oxidized glutathione (GSSG), the plasma was immediately deproteinized with trichloroacetic acid. The plasma hydrogen peroxide levels were assessed in 489 T2D patients, whereas the GSSG levels were measured in 258 diabetics that were recruited at the final study phase (between March 2019 and October 2019). The GSSG levels were determined by a fluorometric assay protocol (GSH/GSSG Ratio Detection Assay Kit II, Abcam, Waltham, MA, USA) that used a proprietary, non-fluorescent, water-soluble dye that became strongly fluorescent upon reacting with GSH. The levels of ROS were quantified by fluorometric assay using the OxiSelect™ In Vitro ROS/RNS Assay Kit (Cell Biolabs, San Diego, CA, USA), which employed a proprietary quenched fluorogenic probe, dichlorodihydrofluorescin DiOxyQ (DCFH-DiOxyQ), which is a specific ROS/RNS probe. It was first primed with a quench removal reagent and subsequently stabilized in a highly reactive DCFH form. In this reactive state, the ROS and RNS species react with the DCFH, which is rapidly oxidized to the highly fluorescent 2’,7’-dichlorodihydrofluorescein. The standard curve of H_2_O_2_ was used to quantify the ROS concentrations in the plasma samples. Absorbance at 405 nm and fluorescence at 480 nm excitation/530 nm emission were measured on a microplate reader Varioscan Flash (Thermo Fisher Scientific, Waltham, MA, USA). The concentrations of glycated hemoglobin, the fasting blood glucose in blood plasma were determined with the use of a semi-automatic biochemical analyzer Clima MC-15 (Ral Tecnica para el Laboratorio, S.A., Barcelona, Spain) and the reagents produced by DIAKON-DS, Moscow, (Russia). These biochemical and genetic analyses were performed at the Research Institute for Genetic and Molecular Epidemiology of Kursk State Medical University, Kursk (Russia).

### 2.4. Statistical and Bioinformatics Analysis

Statistical power was estimated using the genetic association study power calculator (http://csg.sph.umich.edu/abecasis/gas_power_calculator/, accessed on 12 June 2016). Based on the sample size of 1470 people with T2D, a sub-group association analysis of the *RAC1* polymorphisms with diabetic complications could detect a genotype relative risk of 1.25–1.50, assuming a 79.1–90.0% power and a 5% type I error (0.05). The chi-square test was used to compare the genotype frequencies in T2D patients to the values predicted by the Hardy–Weinberg equilibrium assumption. The association between the *RAC1* gene polymorphisms and diabetic combinations was evaluated by a multiple logistic regression analysis, with the calculation of odds ratios (OR) and 95% confidence intervals (95%CI) adjusted for age, sex, and body mass index (BMI). The associations were analyzed using the SNPStats software [24]. A codominant model was used to present the results in tables. A *p*-value of ≤0.05 was selected as statistically significant. To control for the multiple testing of the SNP-phenotype associations, the calculated *p*-values were adjusted by the false discovery rate (FDR). A Q-value of ≤0.05 was considered statistically significant to interpret the genotype–phenotype associations [25].

The Kolmogorov–Smirnov test was used to determine the normality of the biochemical parameters. Age and BMI were expressed as means with standard deviations and compared between the groups by the Student’s *t*-test. The non-normally distributed traits (glycated hemoglobin, fasting blood glucose, hydrogen peroxide, and total glutathione) were expressed as medians with the first and third quartiles (Q1–Q3) and compared between the groups with the Kruskal–Wallis test. These statistical calculations were performed using the STATISTICA for Windows v13.0 package (TIBCO, Palo Alto, CA, USA).

## 3. Results

### 3.1. Demographic, Clinical and Laboratory Characteristics of Patients

The demographic, clinical, and laboratory characteristics of the study patients are shown in Table 1. The majority of the T2D patients had diabetic neuropathy (92.3%) and diabetic retinopathy (71.2%). Other T2D complications included diabetic angiopathy of the lower extremities (65.9%), diabetic nephropathy (38.4%), diabetic foot syndrome (7.6%), and coronary artery disease (32.5%). The patients with the above complications had a significantly longer duration of T2D (*p =* 0.001). As can be seen from Table 1, there were no regularities or trends in the quantitative parameters of redox homeostasis, such as glutathione or hydrogen peroxide, regardless of the type of diabetes complication.

### 3.2. Association of RAC1 Gene Polymorphisms with Diabetic Retinopathy

The frequency of the minor allele rs7784465-C was significantly higher in the patients with DR within the entire group (OR 1.35, 95%CI 1.09–1.68, *p =* 0.006) and in males (OR 1.52, 95%CI 1.05–2.21, *p =* 0.032) after a sex-stratified analysis. The alternative allele rs836478-T was associated with DR in the entire group of patients with DR (OR 1.34, 95%CI 1.14–1.59, *p =* 0.0005), in males (OR 1.51, 95%CI 1.15–1.99, *p =* 0.003), and in females (OR 1.27, 95%CI 1.02–1.57, *p =* 0.03). The genotype frequencies of the studied SNPs in diabetics with and without DR are shown in Table 2.

The genotypes rs7784465-T/C and rs836478-T/T were associated with the risk of diabetic retinopathy in the entire group of diabetics. A sex-stratified association analysis showed that the polymorphisms rs7784465 and rs836478 were associated with an increased risk for DR in males, whereas in females, no difference in the genotype frequencies for these SNPs was seen between the patients with and without DR. The estimated frequencies of the *RAC1* haplotypes in T2D patients with and without DR are shown in Table 3.

The frequency of the haplotypes *H2* rs4724800A-rs7784465C-rs10951982G-rs10238136A-rs836478T-rs9374G and *H3* rs4724800G-rs7784465T-rs10951982A-rs10238136A-rs836478T-rs9374A was significantly higher in the patients with DR. A sex-stratified analysis showed a much stronger association of the haplotype *H2* rs4724800A-rs7784465C-rs10951982G-rs10238136A-rs836478T-rs9374G with DR in diabetic males (OR 2.32, 95CI 1.46–3.67, *p =* 0.0004).

### 3.3. Polymorphisms of the RAC1 Gene and Diabetic Nephropathy

The minor allele rs836478-T was associated with DNF exclusively in males (OR 1.43, 95% CI 1.05–1.96, *p =* 0.025). The genotype frequencies for the studied SNPs in the diabetics with and without DNF are shown in Table 4.

As can be seen from Table 4, the polymorphism rs836478 was associated with the risk of DNF in males in the codominant model. However, the rs836478-C/T-T/T genotypes of *RAC1* were found to be associated with an increased risk of diabetic nephropathy in male diabetics (OR 1.84, 95% CI 1.06–3.19, *p =* 0.024) in the dominant model. The other SNPs of the *RAC1* gene showed no significant associations with a DNF risk. A haplotype analysis (Supplementary Table S1) revealed that none of the *RAC1* haplotypes were associated with diabetic nephropathy.

### 3.4. RAC1 Gene Polymorphisms and the Risk of Diabetic Neuropathy

The frequencies of the minor alleles rs7784465-C (OR 1.80, 95% CI 1.17–2.75, *p =* 0.007) and rs836478-T (OR 1.35, 95% CI 1.02–1.80, *p =* 0.037) were significantly higher in the patients with DN compared to the patients without DN. The allele rs7784465-C was also associated with DN in females (OR 2.02, 95% CI 1.08–3.76, *p =* 0.028). The genotype frequencies of the *RAC1* gene polymorphisms among the T2D patients with and without diabetic neuropathy are given in Table 5.

The genotype rs7784465-T/C of *RAC1* was associated with an increased risk of DN in the entire group of T2D patients and diabetic females. As can be seen from Table 6, the haplotype *H2* rs4724800A-rs7784465C-rs10951982G-rs10238136A-rs836478T-rs9374G and the minor alleles rs7784465-C and rs836478-T were associated with an increased risk of DN in both diabetic males and females. Interestingly, the haplotype *H5* rs4724800G-rs7784465T-rs10951982G-rs10238136A-rs836478C-rs9374G showed an association with an increased risk of DN only in males. Meanwhile, the haplotype *H7* rs4724800A-rs7784465T-rs10951982G-rs10238136T-rs836478T-rs9374G possessed a protective effect against the DN risk in diabetic females.

### 3.5. The Link between RAC1 Gene Polymorphisms to Diabetic Angiopathy of the Lower Extremities and Diabetic Foot Syndrome

The minor allele rs10238136-T was found to be associated with diabetic angiopathy of the lower extremities in females (OR 3.47, 95%CI 1.42–8.46, *p =* 0.004). The genotype frequencies for the *RAC1* gene polymorphisms among the T2D patients with and without diabetic angiopathy of the lower extremities are given in Table 7.

The genotype rs10238136-A/T was associated with an increased risk of DA in the entire group and in diabetic females, whereas the genotype rs7784465-T/C was associated with DA only in males. Meanwhile, the haplotype *H7* rs4724800A-rs7784465T-rs10951982G-rs10238136T-rs836478T-rs9374G (Table 8) showed an association with an increased risk of DA in females.

As can be seen from Supplementary Table S2, none of the studied SNPs of the *RAC1* gene showed an association with the risk of diabetic foot syndrome. However, the minor allele rs10238136-T (OR 3.67, 95%CI 1.48–9.10, *p =* 0.016) and haplotype *H6* rs4724800A-rs7784465T-rs10951982G-rs10238136T-rs836478T-rs9374G (Table 9) were associated with diabetic foot syndrome in males.

### 3.6. The Link between RAC1 Gene Haplotypes and Plasma Parameters of Redox Homeostasis

An analysis of the relationship between the genetic and biochemical parameters of redox homeostasis (Supplementary Table S3) revealed an association of the haplotype rs4724800A-rs7784465T-rs10951982G-rs10238136A-rs836478T-rs9374G with increased levels of ROS in the plasma of diabetics with DR (Diff = 1.02, 95% CI 0.18–1.85, *p =* 0.017), DNF (Diff = 1.14, 95% CI 0.26–2.02, *p =* 0.011) and DN (Diff = 0.90, 95% CI 0.25–1.55, *p =* 0.0069). Moreover, DNF patients carrying the same *RAC1* haplotype had a lower concentration of total plasma glutathione (Diff = −1.72, 95% CI −3.01–−0.44, *p =* 0.0095) compared with the carriers of the reference haplotype rs4724800A-rs7784465T-rs10951982G-rs10238136A-rs836478C-rs9374G.

The haplotype rs4724800A-rs7784465C-rs10951982G-rs10238136A-rs836478C-rs9374G was associated with higher ROS levels in patients with DNF (Diff = 6.82, 95% CI 4.90–8.73, *p* < 0.0001), DN (Diff = 5.73, 95% CI 4.44–7.01, *p* < 0.0001), and DA (Diff = 7.23, 95% CI 5.83–8.62, *p* <0.0001). In patients with DFS, the haplotype rs4724800G-rs7784465C-rs10951982A-rs10238136A-rs836478T-rs9374A was associated with increased ROS (Diff = 4.10, 95% CI 2.52–5.68, *p* < 0.0001), whereas the carriers of the haplotypes rs4724800A-rs7784465C-rs10951982G-rs10238136A-rs836478T-rs9374G (Diff = 1.09, 95% CI 0.29–1.90, *p =* 0.012) and rs4724800G-rs7784465T-rs10951982A-rs10238136A-rs836478T-rs9374A (Diff = 1.03, 95% CI 0.08–1.98, *p =* 0.039) had higher levels of total glutathione in their blood plasma (Supplementary Table S3).

## 4. Discussion

### 4.1. Summary of the Study Findings

**Figure 1:**
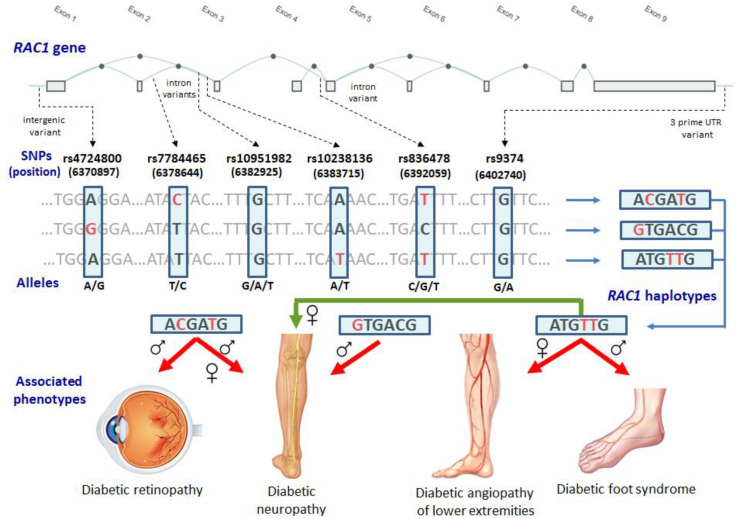
The exon–intron organization of the *RAC1* gene, SNP position, haplotype structure, and a summary of the observed associations.

The present study found, for the first time, that the polymorphisms of the gene encoding Rac family small GTPase 1 (*RAC1*) in type 2 diabetes are associated with complications such as diabetic retinopathy, neuropathy, and angiopathy of the lower extremities. However, the observed associations were sex-specific. In particular, the genotype rs7784465-T/C was associated with an increased risk of retinopathy and angiopathy of the lower extremities in males, as well as diabetic neuropathy in females. Furthermore, the polymorphism rs836478 of *RAC1* was linked to diabetic retinopathy and nephropathy in males, whereas the polymorphism rs10238136 was linked to diabetic angiopathy in females. Figure 1 depicts the structure of the *RAC1* gene, the genomic position of the SNPs, and the haplotype structure of the gene, as well as summarizing the overall research findings. The *RAC1* haplotypes were found to be associated with DR in males and with DN in females. Furthermore, the *RAC1* haplotype rs4724800A-rs7784465C-rs10951982G-rs10238136A-rs836478T-rs9374G showed an association with DR in males and DN regardless of sex. In addition, the haplotype rs4724800A-rs7784465T-rs10951982G-rs10238136T-rs836478T-rs9374G was associated with a 4-fold risk of DA in females and DFS in males. The haplotype rs4724800A-rs7784465T-rs10951982G-rs10238136A-rs836478T-rs9374G showed an association with the increased plasma levels of ROS in diabetics with DR, DNF, and DN. The patients with DNF who carried the above haplotype had lower concentrations of total plasma glutathione. Moreover, the haplotype rs4724800A-rs7784465C-rs10951982G-rs10238136A-rs836478C-rs9374G was associated with higher ROS levels in patients with DNF, DN, and DA. The haplotype rs4724800G-rs7784465C-rs10951982A-rs10238136A-rs836478T-rs9374A was correlated with the increased ROS in patients with DFS, whereas the haplotypes rs4724800A-rs7784465C-rs10951982G-rs10238136A-rs836478T-rs9374G and rs4724800G-rs7784465T-rs10951982A-rs10238136A-rs836478T-rs9374A were correlated with the increased levels of total glutathione in the plasma. A functional annotation of the studied SNPs [20] showed that the minor alleles rs7784465-C, rs10951982-A, rs10238136-T, rs836478-T, and rs9374-A were associated with the increased expression of the *RAC1* gene in various tissues and might be binding sites for transcription factors (TF). For instance, an analysis of the TF-binding affinity of the rs836478 polymorphism (which was associated with DNF) with the atSNP tool [26] (http://atsnp.biostat.wisc.edu/search, accessed on 2 November 2020) has shown that the minor allele rs836478-T was predicted to create binding sites for 34 TFs, including FOXC1, FOXD1, PBX1, GATA3, and POU3F3, which are enriched with GO terms that are related to the development of the nephron epithelium and renal tubules, as assessed by the STRING database [27] (https://string-db.org/, accessed on 14 December 2022).

Although many hypotheses have been proposed to explain the molecular pathways underlying diabetic complications, it is widely accepted that glutathione deficiency, the increased production of reactive oxygen species, and its resulting oxidative stress are the major pathological processes responsible for the development of diabetic complications [28,29,30,31,32].

### 4.2. Diabetic Retinopathy

Diabetic retinopathy is one of the most common complications of diabetes mellitus and is a major global contributor to vision loss and blindness [33,34]. According to a meta-analysis of large, population-based studies, the prevalence and progression of diabetic retinopathy have been linked to the serum levels of HbA1c, total cholesterol, and blood pressure, but only in about 10% of patients with type 2 diabetes [35], suggesting that other factors exist that explain the development of diabetic retinopathy in the majority of diabetics. Numerous studies [18,29,31,36] have shown that oxidative stress plays a key role in the onset of diabetic retinopathy. RAC1 is required for NADPH oxidase 2, an enzyme that generates reactive oxygen species. The transcriptional activity of the *RAC1* gene may be regulated through epigenetic mechanisms. In particular, Kowluru and co-workers observed that the histone mark H3K9me3 at the *Rac1* promoter assists with active DNA methylation-hydroxymethylation reactions, activating *Rac1* gene transcription in diabetic mice [19]. Cells that were exposed to high glucose concentrations were found to exhibit increased signaling in the chain Rac1–Nox2–ROS, increased levels of *Rac1* transcripts, and increased 5-hydroxymethylcytosine levels at the gene promoter [37]. ROS overproduction has been shown to speed up the loss of capillary cells and to cause retinal neurodegeneration through mitochondrial damage, whereas the inhibition of ROS production was found to inhibit caspase-3-mediated neuronal apoptosis and to prevent vision loss [38,39].

### 4.3. Diabetic Nephropathy

Diabetic nephropathy is a clinical syndrome that is characterized by persistent albuminuria and a progressive decline in renal function [40]. DNF is thought to be the most common cause of end-stage renal disease, affecting 20% to 50% of people with diabetes. The mechanisms of DNF are very complex, and despite decades of intensive research, the pathogenesis of this complication in type 2 diabetes is still not fully understood [41,42]. Numerous pathways, processes, molecules, and conditions, such as oxidative stress, the renin-angiotensin-aldosterone system, mitogen-activated protein kinases, the formation of advanced glycosylation end products (AGE), connective tissue growth factor, transforming growth factor beta-1 (TGF-β), and inflammatory cytokines, are known to contribute to the onset and progression of DNF [43,44,45]. The pathways and mediators that are involved in kidney damage in type 2 diabetes share a lot of overlaps. For instance, it has been discovered that oxidative stress damages the kidneys through the activation of the renin-angiotensin-aldosterone system, whereas angiotensin-II itself is capable of causing renal injury through oxidative stress [42]. Another example is NADPH oxidase stimulating the production of TGF-β, which stimulates the production of ROS via NADPH oxidase activation [41]. The experimental observation that the inhibition of oxidative stress improved a renal feature associated with streptozotocin-induced DNF has highlighted the role of oxidative stress in the induction and progression of DNF [46,47]. Meanwhile, oxidative stress can damage cells indirectly by activating other pathological pathways which damage the renal cells through unknown mechanisms [48]. Metabolic and hemodynamic alterations in the kidneys are also linked to oxidative stress, and both have additive detrimental effects on the organ [49].

The direct and indirect mechanisms by which oxidative stress causes kidney damage in diabetes have been proposed. ROS were found to cause direct damage to podocytes, mesangial cells, and endothelial cells, leading to proteinuria and tubule-interstitial fibrosis [50]. The mechanism of this damage was argued to be that the glomerulus, the filtering unit of the kidney, is more sensitive to oxidative injury than the other parts of the nephron [51]. Hyperglycemia is known to induce ROS production and oxidative damage to DNA, lipids, and proteins [52]. Chronic hyperglycemia can cause oxidative stress by increasing angiotensin-II levels, activating protein kinases, and increasing TGF-β expression [53]. For instance, increased angiotensin-II levels induce ROS production in the kidneys through the activation of NADPH oxidase [54]. It is observed that the ROS-associated activation of TGF-β causes the excessive remodeling of the extracellular matrix in the mesangium and promotes fibrotic processes in the kidneys [55]. As mentioned above, the increased production of ROS via NADPH oxidase in diabetes is attributed to the activation of the NF-κB pathway, which also promotes the transcriptional activation of the genes encoding inflammatory cytokines, thereby contributing to kidney injury and leading to renal fibrosis and a decline in renal function [56,57,58]. The activation of the α and β isoforms of protein kinases C is also known to induce oxidative damage to the kidneys through the increased production of NADPH-dependent superoxide anion radicals [59]. There are many other redox-sensitive signal transduction pathways, such as c-Jun N-terminal kinase (JNK), p38 MAP kinase, and the transcription factor activator protein 1 (AP-1), determining a vicious cycle between inflammation and oxidative stress [60,61].

We have established, for the first time, an association between the minor allele rs836478-T and an increased risk of diabetic nephropathy. According to the GTEx portal (https://gtexportal.org, accessed on 24 February 2023), the *RAC1* gene is expressed at a relatively high level in the kidneys, suggesting an important role of the Rac family small GTPase 1 in this organ. There have been no studies on humans or animals that have investigated the expression level of the *RAC1* gene in diabetic nephropathy, but there are studies that have investigated other NOX enzymes. In particular, an increased NOX-4 expression in renal cells was discovered in streptozotocin-induced diabetic rats [62], and subsequent studies have argued that up-regulated NOX-4 is the primary source of the increased ROS production in the kidneys that contributes to renal fibrosis and DNF [63]. Both the deletion and the inhibition of the NOX4 and NOX1 genes have been shown to be renoprotective [64]. Finally, Ying and co-workers have recently observed that the binding of RAC1 to the pyrin domain containing 3 (NLRP3) activates the NLRP3 inflammasome in the kidney and accelerates the pathological processes underlying diabetic nephropathy [65]. The above studies clearly demonstrate the importance of RAC1-mediated oxidative stress for the development of diabetic nephropathy.

### 4.4. Diabetic Angiopathy of Lower Extremities

Diabetic angiopathy of the lower extremities is a change in the structure of the vessels of the legs in patients with diabetes mellitus, in the form of a decrease in the elasticity of the vascular wall and its thickening, leading to the narrowing of the lumen or the complete obliteration of the arteries. Increased oxidative stress is implicated in the pathogenesis of the various vascular complications of diabetes, including in diabetic angiopathy of the lower extremities [66,67,68]. It is well-known that abnormal endothelial-dependent vasodilation in diabetic patients is at least partially attributed to the reactive oxygen species that are primarily generated by up-regulated NOXs and downregulated endothelial nitric oxide synthase [69,70]. The increase in ROS levels and the decrease in nitric oxide are known to cause irreversible damage to the vascular endothelial cells through apoptosis [68]. The increased expression of NOX subunits, such as p22phox, p47phox, and p67phox, and the associated increased production of vascular superoxide anion radicals have been identified in diabetic subjects [71].

### 4.5. Diabetic Neuropathy

Diabetic neuropathy is a unique neurodegenerative disorder of the peripheral nervous system that preferentially targets sensory axons, autonomic axons, and later, to a lesser extent, motor axons [72]. The peripheral neurons that supply the feet are the longest cells in the body and require a properly functioning vascular supply, mitochondria, and glucose and lipid metabolism [73]. The duration of the diabetes and the plasma levels of the HBA1c are considered to be major predictors of diabetic neuropathy [74]. We revealed an association of the genotype rs7784465-T/C of the *RAC1* gene with an increased risk of diabetic neuropathy in females. Female sex was found to be a risk factor for painful diabetic neuropathy, which is consistent with our findings [75]. The overproduction of superoxide anions has even been implicated in diabetic microvascular complications [76]. ROS production inhibits the GAPDH enzyme (glyceraldehyde-3-phosphate dehydrogenase) activity, causing upstream glycolytic metabolites to be diverted into the molecular pathways of glucose overutilization [77]. It is known that ROS production overwhelms the endogenous antioxidant defense in diabetic peripheral neuropathy, impairing the neural blood flow, nerve conduction, neurotrophic support, and neuronal mitochondrial function [78,79]. Hyperglycemia-induced oxidative and/or nitrosative stress causes DNA damage and the subsequent hyperactivation of poly(ADP-ribose) polymerases (PARP), which are the enzymes involved in DNA repair, cellular proliferation, and programmed cell death [80]. Overactivated PARPs consume NAD+, slowing glycolysis and impairing ATP function, as well as inhibiting GAPDH. PARP activation also promotes the formation of excess amounts of the superoxide anions and peroxynitrites that are associated with endothelial dysfunction, decreased nerve blood flow, neuronal energy deficit, a loss of nerve fiber density, and nerve conduction slowing [81,82].

### 4.6. Diabetic Foot Syndrome

Diabetic foot syndrome is a long-term complication of type 2 diabetes that is caused by a combination of vascular and neurological deterioration [83]. Epidemiological studies have shown that neuropathy is responsible for about 50% of the cases of diabetic foot syndrome [84]. Our study revealed that the *RAC1* haplotype rs4724800A-rs7784465T-rs10951982G-rs10238136T-rs836478T-rs9374G was associated with a four-fold risk of DFS in males. The study of Rossboth S. and co-workers found a positive association of DFS with the male sex [85]. The pathogenesis of DFS has been linked to a variety of conditions, including oxidative stress, the malfunction of polyol and inositol metabolism, increased Na/K-ATPase activity, endoneural microvascular deficits and ischemia, defective axonal transport, and the non-enzymatic glycosylation of proteins in peripheral neurons [86,87].

The study has some limitations. Because the sample size of the patients with a specific diabetic complication was relatively small, the statistical power of the association analysis that was performed in the subgroups was decreased. A limited number of patients undergoing biochemical investigations of their redox homeostasis did not allow for the obtainment of more reliable estimates of the effects of the studied SNPs on these parameters in subgroups with particular diabetic complications. This limitation made it difficult to interpret the revealed associations between the *RAC1* haplotypes and the plasma levels of the ROS and total glutathione. Furthermore, there may be other unexplored confounding variables in the diabetics that contribute to the development of diabetic complications.

## 5. Conclusions

The present study demonstrated, for the first time, that the genetic variants in the *RAC1* gene represent novel susceptibility markers for diabetic retinopathy, nephropathy, angiopathy of the lower extremities, and neuropathy, with the potential to influence the risk of diabetic complications through perturbations in redox homeostasis. The sexual dimorphism of the associations between the *RAC1* gene polymorphisms and the risk of diabetic retinopathy, particularly in men, appears to be due to the male sex itself being a known risk factor for this complication [85,88]. The mechanisms underlying the sex-specific associations of these genetic polymorphisms with a susceptibility for common diseases are a hallmark of research and continue to pique the interest of scientists [89,90]. The associations of the *RAC1* gene haplotypes with the elevated concentrations of reactive oxygen species in patients with diabetic retinopathy, nephropathy, neuropathy, angiopathy, and diabetic foot syndrome may be intermediate damaging factors underlying the development of microvascular and nerve tissue diabetic complications. Because this is the first study to look into the role of the *RAC1* gene polymorphisms in diabetic complications, there are no comparable studies to compare our findings to. Further studies into other populations of the world are required to validate these associations between the polymorphisms of the *RAC1* gene and diabetic complications. However, our findings can already be applied to the development of new pharmacological agents that inhibit the RAC1 expression in specific tissues and thus reduce the ROS production.

## Figures and Tables

**Table 1:** Demographic, clinical, and biochemical characteristics of the study patients.

Baseline Characteristics	T2D Patients (*n* = 1470)		*p*-Value *
	Without Complication	With Complication	
Diabetic retinopathy			
Sample size, *n* (%)	407 (28.8)	1007 (71.2)	*-*
Age, mean ± SD	58.50 ± 11.73	62.99 ± 9.37	**<0.0001**
Males, *n* (%)	179 (44.0)	279 (27.7)	**<0.0001**
Females, *n* (%)	228 (56.0)	728 (72.3)	
Body mass index (kg/m^2^), mean ± SD	31.65 ± 6.91	32.57 ± 6.55	0.18
Duration of diabetes, median (Q1;Q3)	2.0 (1.0; 8.0)	10.0 (6.0; 16.0)	**0.001**
HbA_1C_ (%), Me (Q1; Q3)	9.3 (7.9; 11.1)	9.0 (7.7; 10.6)	**0.033**
FBG (mmol/L), Me (Q1; Q3)	12.8 (10.3; 15.8)	12.0 (9.53; 15.0)	**0.001**
H_2_O_2_ (mmol/L), Me(Q1;Q3)	3.94 (2.70; 5.27)	3.66 (2.58; 4.92)	0.11
GSSG/GSH (mmol/L), Me(Q1;Q3)	2.70 (1.29; 3.90)	1.28 (0.50; 3.74)	**0.0036**
Diabetic nephropathy			
Sample size, *n* (%)	887 (61.6)	553 (38.4)	-
Age, mean ± SD	58.86 ± 10.29	66.11 ± 8.60	**<0.0001**
Males, *n* (%)	379 (42.7)	101 (18.3)	**<0.0001**
Females, *n* (%)	507 (57.3)	452 (81.7)	
Body mass index (kg/m^2^), mean ± SD	31.62 ± 6.88	33.15 ± 6.29	**<0.0001**
Duration of diabetes, median (Q1;Q3)	7.0 (2.0; 12.0)	11.0 (6.0; 17.0)	**<0.0001**
HbA_1C_ (%), Me (Q1; Q3)	9.0 (7.7; 10.5)	9.2 (7.8; 11.0)	0.09
FBG (mmol/L), Me (Q1; Q3)	12.0 (9.5; 15.0)	12.6 (10.0; 15.9)	**0.026**
H_2_O_2_ (mmol/L), Me(Q1;Q3)	3.73 (2.62; 5.09)	3.74 (2.66; 4.99)	0.93
GSSG/GSH (mmol/L), Me(Q1;Q3)	1.31 (0.56; 3.77)	1.99 (0.56; 3.90)	0.29
Diabetic neuropathy			
Sample size, *n* (%)	109 (7.7)	1309 (92.3)	-
Age, mean ± SD	53.66 ± 12.99	62.37 ± 9.76	**<0.0001**
Males, *n* (%)	58 (53.21)	401 (30.63)	**<0.0001**
Females, *n* (%)	50 (46.79)	908 (69.37)	
Body mass index (kg/m^2^), mean ± SD	29.30 ± 6.24	32.57 ± 6.63	**<0.0001**
Duration of diabetes, median (Q1;Q3)	1.0 (0.1; 1.0)	10.0 (4.0; 15.0)	**0.001**
HbA_1C_ (%), Me (Q1; Q3)	9.4 (7.7; 11.3)	9.0 (7.7; 10.8)	0.18
FBG (mmol/L), Me (Q1; Q3)	13.1 (10.0)	12.2 (9.8; 15.2)	0.14
H_2_O_2_ (mmol/L), Me(Q1;Q3)	3.98 (3.21; 6.41)	3.72 (2.6; 4.94)	**0.013**
GSSG/GSH (mmol/L), Me(Q1;Q3)	1.31 (0.29; 3.66)	1.64 (0.57; 3.80)	0.55
Angiopathy of the lower extremities			
Sample size, *n* (%)	502 (34.1)	968 (65.9)	-
Age, mean ± SD	58.33 ± 12.09	63.19 ± 9.27	**<0.0001**
Males, *n* (%)	210 (41.8)	272 (28.1)	**<0.0001**
Females, *n* (%)	291 (58.2)	696 (71.9)	
Body mass index (kg/m^2^), mean ± SD	31.12 ± 6.51	32.81 ± 6.65	**<0.0001**
Duration of diabetes, median (Q1;Q3)	5.0 (1.0; 11.0)	10.0 (5.0; 15.0)	**<0.0001**
HbA_1C_ (%), Me (Q1; Q3)	9.2 (7.75; 11.2)	9.0 (7.7; 10.5)	0.054
FBG (mmol/L), Me (Q1; Q3)	12.5 (9.7; 15.6)	12.0 (9.79; 15.0)	0.23
H_2_O_2_ (mmol/L), Me(Q1;Q3)	3.70 (2.76; 5.12)	3.72 (2.58; 5.02)	0.58
GSSG/GSH (mmol/L), Me(Q1;Q3)	1.35 (0.56; 3.82)	1.70 (0.51; 3.78)	0.66
Diabetic foot syndrome			
Sample size, *n* (%)	1309 (92.4)	107 (7.6)	-
Age, mean ± SD	61.57 ± 10.45	63.35 ± 8.29	0.087
Males, *n* (%)	424 (32.4)	34 (31.8)	0.89
Females, *n* (%)	884 (67.6)	73 (68.2)	
Body mass index (kg/m^2^), mean ± SD	32.27 ± 6.67	33.02 ± 6.65	0.26
Duration of diabetes, median (Q1;Q3)	9.0 (3.0; 14.0)	12.0 (6.0; 18.0)	**0.0002**
HbA_1C_ (%), Me (Q1; Q3)	9.1 (7.7; 10.9)	8.4 (7.5; 10.0)	**0.032**
FBG (mmol/L), Me (Q1; Q3)	12.4 (9.88; 15.32)	10.9 (8.86; 14.3)	**0.012**
H_2_O_2_ (mmol/L), Me(Q1;Q3)	3.81 (2.73; 5.14)	2.72 (1.95; 4.03)	**0.0003**
GSSG/GSH (mmol/L), Me(Q1;Q3)	1.41 (0.51; 3.71)	2.83 (1.23; 3.98)	0.051

* Bold is statistically significant *p*-value.

**Table 2:** Genotype frequencies for the *RAC1* gene polymorphisms in T2D patients with and without diabetic retinopathy (DR).

SNP	Genotype	T2D Patients		*P* (*Q*) ^1^	OR (95% CI) ^2^
		Without DR *n* (%)	With DR *n* (%)		
Entire Group					
rs4724800 A>G	A/A	253 (62.9)	581 (58.2)	0.14 (0.33)	1.00
	A/G	127 (31.6)	370 (37.1)		1.28 (0.99–1.65)
	G/G	22 (5.5)	47 (4.7)		0.93 (0.54–1.61)
rs7784465 T>C	T/T	280 (70.5)	618 (63.4)	**0.034** (0.15)	1.00
	T/C	104 (26.2)	317 (32.5)		**1.36 (1.04–1.77)**
	C/C	13 (3.3)	40 (4.1)		1.39 (0.72–2.69)
rs10951982 G>A	G/G	272 (67.5)	624 (62.6)	0.19 (0.38)	1.00
	G/A	112 (27.8)	329 (33)		1.27 (0.98–1.66)
	A/A	19 (4.7)	43 (4.3)		1.00 (0.56–1.79)
rs10238136 A>T	A/A	374 (95.2)	935 (94.2)	0.51 (0.55)	1.00
	A/T	18 (4.6)	57 (5.7)		1.31 (0.75–2.28)
	T/T	1 (0.2)	1 (0.1)		0.40 (0.02–6.43)
rs836478 C>T	C/C	144 (35.6)	278 (28.4)	**0.0013** **(0.023)**	1.00
	C/T	194 (48)	469 (48)		1.30 (0.99–1.70)
	T/T	66 (16.3)	231 (23.6)		**1.89 (1.33–2.69)**
rs9374 G>A	G/G	275 (67.6)	614 (63)	0.21 (0.38)	1.00
	G/A	116 (28.5)	324 (33.3)		1.26 (0.97–1.64)
	A/A	16 (3.9)	36 (3.7)		1.02 (0.54–1.92)
Males					
rs4724800 A>G	A/A	111 (63.1)	163 (59.5)	0.42 (0.52)	1.00
	A/G	53 (30.1)	96 (35)		1.28 (0.84–1.97)
	G/G	12 (6.8)	15 (5.5)		0.84 (0.37–1.91)
rs7784465 T>C	T/T	129 (75.4)	167 (63)	**0.016** (0.096)	1.00
	T/C	37 (21.6)	92 (34.7)		**1.93 (1.22–3.06)**
	C/C	5 (2.9)	6 (2.3)		1.11 (0.32–3.83)
rs10951982 G>A	G/G	121 (68.4)	176 (63.8)	0.28 (0.46)	1.00
	G/A	44 (24.9)	87 (31.5)		1.37 (0.88–2.15)
	A/A	12 (6.8)	13 (4.7)		0.79 (0.34–1.86)
rs10238136 A>T	A/A	161 (94.7)	257 (93.1)	0.14 (0.34)	1.00
	A/T	8 (4.7)	19 (6.9)		1.67 (0.69–4.05)
	T/T	1 (0.6)	0 (0)		NA
rs836478 C>T	C/C	63 (35.4)	71 (26.4)	**0.0051** (**0.046**)	1.00
	C/T	89 (50)	128 (47.6)		1.38 (0.88–2.18)
	T/T	26 (14.6)	70 (26)		**2.58 (1.43–4.63)**
rs9374 G>A	G/G	122 (68.2)	169 (62.8)	0.15 (0.34)	1.00
	G/A	46 (25.7)	90 (33.5)		1.43 (0.92–2.22)
	A/A	11 (6.2)	10 (3.7)		0.66 (0.26–1.67)
Females					
rs4724800 A>G	A/A	142 (62.8)	418 (57.7)	0.31 (0.47)	1.00
	A/G	74 (32.7)	274 (37.9)		1.28 (0.93–1.77)
	G/G	10 (4.4)	32 (4.4)		1.03 (0.49–2.17)
rs7784465 T>C	T/T	151 (66.8)	451 (63.5)	0.52 (0.55)	1.00
	T/C	67 (29.6)	225 (31.7)		1.12 (0.80–1.56)
	C/C	8 (3.5)	34 (4.8)		1.49 (0.67–3.32)
rs10951982 G>A	G/G	151 (66.8)	448 (62.2)	0.43 (0.52)	1.00
	G/A	68 (30.1)	242 (33.6)		1.22 (0.88–1.69)
	A/A	7 (3.1)	30 (4.2)		1.32 (0.56–3.09)
rs10238136 A>T	A/A	213 (95.5)	678 (94.6)	0.70 (0.70)	1.00
	A/T	10 (4.5)	38 (5.3)		1.14 (0.56–2.33)
	T/T	0 (0)	1 (0.1)		NA
rs836478 C>T	C/C	81 (35.8)	207 (29.2)	0.098 (0.34)	1.00
	C/T	105 (46.5)	341 (48.1)		1.28 (0.91–1.79)
	T/T	40 (17.7)	161 (22.7)		1.59 (1.03–2.46)
rs9374 G>A	G/G	153 (67.1)	445 (63.1)	0.40 (0.52)	1.00
	G/A	70 (30.7)	234 (33.2)		1.17 (0.85–1.63)
	A/A	5 (2.2)	26 (3.7)		1.65 (0.62–4.39)

^1^ *p*-value (FDR-adjusted *p*-value) adjusted for age, sex, and BMI. ^2^ Odds ratio with 95% confidence intervals adjusted for age, sex, and BMI (codominant genetic model). Bold is statistically significant *P*- and *Q*-values.

**Table 3:** Estimated common haplotype frequencies of *RAC1* gene in T2D patients with and without diabetic retinopathy.

Haplotype Number	SNPs						T2D Patients		OR (95% CI) ^2^	*P* (Q) ^3^
	rs4724800	rs7784465	rs10951982	rs10238136	rs836478	rs9374				
							Without DR	With DR		
							Haplotype Frequency ^1^			
Entire group Global haplotype association *p*-value: 0.077										
*H1*	A	T	G	A	C	G	0.5215	0.4644	1.00	---
*H2*	A	C	G	A	T	G	0.1457	0.1878	**1.46 (1.14–1.87)**	**0.003** (**0.034**)
*H3*	G	T	A	A	T	A	0.1572	0.1755	**1.30 (1.02–1.66)**	**0.036** (0.28)
*H4*	A	T	G	A	T	G	0.0733	0.0762	1.25 (0.89–1.77)	0.20 (0.46)
*H5*	G	T	G	A	C	G	0.0290	0.0221	0.94 (0.54–1.63)	0.81 (0.93)
*H6*	G	T	A	A	C	A	0.0220	0.0202	1.05 (0.55–2.03)	0.88 (0.96)
*H7*	A	T	G	T	T	G	0.0181	0.0168	1.30 (0.66–2.56)	0.45 (0.74)
*rare*	*	*	*	*	*	*	0.0152	0.0063	1.18 (0.70–1.99)	0.54 (0.78)
Males Global haplotype association *p*-value: **0.009**										
*H1*	A	T	G	A	C	G	0.5326	0.4438	1.00	---
*H2*	A	C	G	A	T	G	0.111	0.1839	**2.32 (1.46–3.67)**	**0.0004** (**0.009**)
*H3*	G	T	A	A	T	A	0.1732	0.1689	1.14 (0.78–1.66)	0.50 (0.77)
*H4*	A	T	G	A	T	G	0.0800	0.1033	1.50 (0.90–2.51)	0.12 (0.39)
*H5*	G	T	G	A	C	G	0.0253	0.0213	1.03 (0.41–2.56)	0.95 (0.96)
*H6*	G	T	A	A	C	A	0.0138	0.0220	2.08 (0.63–6.90)	0.23 (0.48)
*H7*	A	T	G	T	T	G	0.0249	0.0240	1.13 (0.48–2.70)	0.78 (0.93)
*H8*	A	C	G	A	C	G	0.0241	0.0056	0.20 (0.04–1.03)	0.06 (0.29)
*rare*	*	*	*	*	*	*	0.0033	0.0030	2.19 (0.74–6.49)	0.16 (0.46)
Females Global haplotype association *p*-value: 0.22										
*H1*	A	T	G	A	C	G	0.4478	0.4745	1.00	---
*H2*	A	C	G	A	T	G	0.1659	0.1791	1.01 (0.74–1.38)	0.96 (0.96)
*H3*	G	T	A	A	T	A	0.1956	0.1582	0.75 (0.56–1.02)	0.07 (0.29)
*H4*	A	T	G	A	T	G	0.0765	0.0734	0.88 (0.56–1.39)	0.58 (0.78)
*H5*	G	T	G	A	C	G	0.0349	0.0203	0.56 (0.29–1.09)	0.09 (0.33)
*H6*	G	T	A	A	C	A	0.0244	0.0268	0.85 (0.37–1.92)	0.69 (0.88)
*H7*	A	T	G	T	T	G	0.0059	0.0180	0.57 (0.21–1.55)	0.27 (0.52)
*H8*	A	C	G	A	C	G	0.0195	0.0135	2.38 (0.63–9.02)	0.20 (0.46)
*rare*	*	*	*	*	*	*	0.0121	0.0063	1.49 (0.66–3.38)	0.34 (0.60)

^1^ Rare haplotypes with frequency < 0.01 are not shown and indicated as “*”. ^2^ Odds ratio with 95% confidence intervals adjusted for age, sex, and BMI (codominant genetic model). ^3^ *p*-value (FDR-adjusted *p*-value) adjusted for age, sex, and BMI. Bold is statistically significant *P*- and *Q*-values.

**Table 4:** Genotype frequencies for the studied gene polymorphisms among T2D patients with and without diabetic nephropathy.

*RAC1* SNP ID	Genotype	T2D Patients		*P* (*Q*) ^1^	OR (95% CI) ^2^
		Without DNF *n* (%)	With DNF *n* (%)		
Entire group					
rs4724800 A>G	A/A	520 (59.5)	322 (58.4)	0.42 (0.74)	1.00
	A/G	315 (36)	197 (35.8)		1.04 (0.82–1.33)
	G/G	39 (4.5)	32 (5.8)		1.44 (0.84–2.47)
rs7784465 T>C	T/T	565 (65.8)	347 (64.6)	0.99 (0.99)	1.00
	T/C	261 (30.4)	169 (31.5)		1.00 (0.78–1.30)
	C/C	33 (3.8)	21 (3.9)		1.04 (0.56–1.91)
rs10951982 G>A	G/G	557 (63.7)	346 (63)	0.31 (0.74)	1.00
	G/A	284 (32.5)	173 (31.5)		0.99 (0.77–1.27)
	A/A	33 (3.8)	30 (5.5)		1.57 (0.88–2.81)
rs10238136 A>T	A/A	818 (94.1)	515 (95)	0.66 (0.85)	1.00
	A/T	50 (5.8)	26 (4.8)		0.81 (0.48–1.37)
	T/T	1 (0.1)	1 (0.2)		1.84 (0.11–30.29)
rs836478 C>T	C/C	262 (30.5)	162 (29.6)	0.60 (0.83)	1.00
	C/T	418 (48.7)	260 (47.5)		1.04 (0.79–1.36)
	T/T	179 (20.8)	125 (22.9)		1.18 (0.85–1.63)
rs9374 G>A	G/G	544 (63.5)	352 (64.2)	0.17 (0.74)	1.00
	G/A	286 (33.4)	169 (30.8)		0.94 (0.73–1.21)
	A/A	27 (3.1)	27 (4.9)		1.74 (0.93–3.25)
Males					
rs4724800 A>G	A/A	218 (58.8)	62 (61.4)	0.91 (0.96)	1.00
	A/G	130 (35)	33 (32.7)		0.90 (0.55–1.48)
	G/G	23 (6.2)	6 (5.9)		0.91 (0.34–2.43)
rs7784465 T>C	T/T	246 (68.5)	62 (63.3)	0.56 (0.83)	1.00
	T/C	103 (28.7)	34 (34.7)		1.32 (0.80–2.16)
	C/C	10 (2.8)	2 (2)		1.03 (0.21–5.01)
rs10951982 G>A	G/G	237 (63.4)	66 (66)	0.40 (0.74)	1.00
	G/A	118 (31.6)	27 (27)		0.78 (0.47–1.32)
	A/A	19 (5.1)	7 (7)		1.50 (0.58–3.90)
rs10238136 A>T	A/A	346 (94)	93 (93)	0.28 (0.74)	1.00
	A/T	22 (6)	6 (6)		1.15 (0.44–3.03)
	T/T	0 (0)	1 (1)		NA
rs836478 C>T	C/C	115 (31.2)	21 (21.2)	0.056 (0.74)	1.00
	C/T	179 (48.5)	50 (50.5)		1.70 (0.95–3.04)
	T/T	75 (20.3)	28 (28.3)		**2.16 (1.12–4.17)**
rs9374 G>A	G/G	233 (63)	64 (64.7)	0.29 (0.74)	1.00
	G/A	121 (32.7)	28 (28.3)		0.80 (0.47–1.33)
	A/A	16 (4.3)	7 (7.1)		1.81 (0.67–4.86)
Females					
rs4724800 A>G	A/A	302 (60)	260 (57.8)	0.22 (0.74)	1.00
	A/G	185 (36.8)	164 (36.4)		1.10 (0.83–1.46)
	G/G	16 (3.2)	26 (5.8)		1.80 (0.91–3.57)
rs7784465 T>C	T/T	319 (63.8)	285 (64.9)	0.82 (0.92)	1.00
	T/C	158 (31.6)	135 (30.8)		0.91 (0.68–1.23)
	C/C	23 (4.6)	19 (4.3)		1.02 (0.52–1.99)
rs10951982 G>A	G/G	320 (64)	280 (62.4)	0.45 (0.74)	1.00
	G/A	166 (33.2)	146 (32.5)		1.07 (0.80–1.44)
	A/A	14 (2.8)	23 (5.1)		1.57 (0.76–3.25)
rs10238136 A>T	A/A	472 (94.2)	422 (95.5)	0.32 (0.74)	1.00
	A/T	28 (5.6)	20 (4.5)		0.71 (0.38–1.31)
	T/T	1 (0.2)	0 (0)		NA
rs836478 C>T	C/C	147 (30)	141 (31.5)	0.77 (0.92)	1.00
	C/T	239 (48.8)	210 (46.9)		0.90 (0.65–1.23)
	T/T	104 (21.2)	97 (21.6)		0.97 (0.66–1.42)
rs9374 G>A	G/G	311 (63.9)	288 (64.1)	0.44 (0.74)	1.00
	G/A	165 (33.9)	141 (31.4)		0.99 (0.74–1.33)
	A/A	11 (2.3)	20 (4.5)		1.67 (0.75–3.73)

^1^ *p*-value (FDR-adjusted *p*-value) adjusted for age, sex, and BMI. ^2^ Odds ratio with 95% confidence intervals adjusted for age, sex, and BMI (codominant genetic model). Bold is statistically significant *P*- and *Q*-values.

**Table 5:** Genotype frequencies for the studied gene polymorphisms among T2D patients with and without diabetic neuropathy.

*RAC1* SNP ID	Genotype	T2D Patients		*P* (*Q*) ^1^	OR (95% CI) ^2^
		Without DN *n* (%)	With DN *n* (%)		
Entire group					
rs4724800 A>G	A/A	69 (65.1)	766 (59.1)	0.62 (0.73)	1.00
	A/G	33 (31.1)	466 (35.9)		1.20 (0.76–1.87)
	G/G	4 (3.8)	65 (5)		1.44 (0.49–4.26)
rs7784465 T>C	T/T	83 (79)	817 (64.3)	**0.008** (0.13)	1.00
	T/C	19 (18.1)	403 (31.7)		**2.16 (1.27–3.67)**
	C/C	3 (2.9)	50 (3.9)		1.99 (0.57–6.96)
rs10951982 G>A	G/G	74 (69.2)	821 (63.4)	0.49 (0.73)	1.00
	G/A	27 (25.2)	418 (32.3)		1.29 (0.80–2.07)
	A/A	6 (5.6)	56 (4.3)		0.83 (0.33–2.11)
rs10238136 A>T	A/A	93 (91.2)	1219 (94.7)	0.29 (0.54)	1.00
	A/T	9 (8.8)	66 (5.1)		0.53 (0.24–1.14)
	T/T	0 (0)	2 (0.2)		NA
rs836478 C>T	C/C	40 (37.4)	380 (29.7)	0.097 (0.44)	1.00
	C/T	51 (47.7)	616 (48.2)		1.36 (0.86–2.15)
	T/T	16 (14.9)	282 (22.1)		1.94 (1.04–3.64)
rs9374 G>A	G/G	75 (69.4)	813 (63.7)	0.65 (0.73)	1.00
	G/A	29 (26.9)	415 (32.5)		1.24 (0.78–1.97)
	A/A	4 (3.7)	48 (3.8)		1.15 (0.38–3.47)
Males					
rs4724800 A>G	A/A	39 (68.4)	235 (59.6)	0.18 (0.46)	1.00
	A/G	17 (29.8)	133 (33.8)		1.29 (0.68–2.45)
	G/G	1 (1.8)	26 (6.6)		4.76 (0.58–38.95)
rs7784465 T>C	T/T	42 (76.4)	254 (66.5)	0.33 (0.54)	1.00
	T/C	12 (21.8)	118 (30.9)		1.61 (0.79–3.29)
	C/C	1 (1.8)	10 (2.6)		2.20 (0.26–19.02)
rs10951982 G>A	G/G	40 (70.2)	256 (64.5)	0.57 (0.73)	1.00
	G/A	15 (26.3)	118 (29.7)		1.17 (0.60–2.28)
	A/A	2 (3.5)	23 (5.8)		2.12 (0.44–10.23)
rs10238136 A>T	A/A	51 (92.7)	368 (93.9)	0.93 (0.93)	1.00
	A/T	4 (7.3)	23 (5.9)		0.87 (0.27–2.84)
	T/T	0 (0)	1 (0.3)		NA
rs836478 C>T	C/C	21 (36.2)	111 (28.5)	0.095 (0.44)	1.00
	C/T	30 (51.7)	189 (48.5)		1.39 (0.72–2.66)
	T/T	7 (12.1)	90 (23.1)		2.74 (1.05–7.15)
rs9374 G>A	G/G	40 (69)	250 (63.9)	0.32 (0.54)	1.00
	G/A	17 (29.3)	121 (30.9)		1.06 (0.56–2.02)
	A/A	1 (1.7)	20 (5.1)		3.99 (0.47–33.53)
Females					
rs4724800 A>G	A/A	30 (61.2)	531 (58.8)	0.69 (0.73)	1.00
	A/G	16 (32.6)	333 (36.9)		1.12 (0.59–2.11)
	G/G	3 (6.1)	39 (4.3)		0.62 (0.18–2.17)
rs7784465 T>C	T/T	41 (82)	563 (63.4)	**0.014** (0.13)	1.00
	T/C	7 (14)	285 (32.1)		**3.01 (1.31–6.91)**
	C/C	2 (4)	40 (4.5)		1.88 (0.41–8.72)
rs10951982 G>A	G/G	34 (68)	565 (62.9)	0.14 (0.46)	1.00
	G/A	12 (24)	300 (33.4)		1.44 (0.72–2.85)
	A/A	4 (8)	33 (3.7)		0.39 (0.13–1.20)
rs10238136 A>T	A/A	42 (89.4)	851 (95.1)	0.16 (0.46)	1.00
	A/T	5 (10.6)	43 (4.8)		0.34 (0.12–0.93)
	T/T	0 (0)	1 (0.1)		NA
rs836478 C>T	C/C	19 (38.8)	269 (30.3)	0.55 (0.73)	1.00
	C/T	21 (42.9)	427 (48.1)		1.38 (0.72–2.66)
	T/T	9 (18.4)	192 (21.6)		1.45 (0.63–3.34)
rs9374 G>A	G/G	35 (70)	563 (63.6)	0.23 (0.52)	1.00
	G/A	12 (24)	294 (33.2)		1.48 (0.75–2.93)
	A/A	3 (6)	28 (3.2)		0.46 (0.13–1.64)

^1^ *p*-value (FDR-adjusted *p*-value) adjusted for age, sex, and BMI. ^2^ Odds ratio with 95% confidence intervals adjusted for age, sex, and BMI (codominant genetic model). Bold is statistically significant *P*- and *Q*-values.

**Table 6:** Estimated common haplotype frequencies of *RAC1* gene in T2D patients with and without diabetic neuropathy.

Haplotype Number	SNPs						T2D Patients		OR (95% CI) ^2^	*P* (Q) ^3^
	rs4724800	rs7784465	rs10951982	rs10238136	rs836478	rs9374				
							Without DN	With DN		
							Haplotype Frequency ^1^			
Entire group Global haplotype association *p*-value: **0.036**										
*H1*	A	T	G	A	C	G	0.5517	0.4750	1.00	---
*H2*	A	C	G	A	T	G	0.0999	0.1815	**2.22 (1.34–3.68)**	**0.0019 (0.02)**
*H3*	G	T	A	A	T	A	0.1608	0.1713	1.20 (0.78–1.82)	0.41 (0.72)
*H4*	A	T	G	A	T	G	0.0889	0.0744	1.11 (0.63–1.94)	0.72 (0.95)
*H5*	G	T	G	A	C	G	0.0178	0.0237	1.37 (0.45–4.15)	0.58 (0.81)
*H6*	G	T	A	A	C	A	0.0105	0.0220	2.53 (0.59–10.85)	0.21 (0.60)
*H7*	A	T	G	T	T	G	0.0344	0.0155	0.58 (0.23–1.42)	0.23 (0.60)
*rare*	*	*	*	*	*	*	0.0106	0.0085	0.95 (0.40–2.26)	0.90 (0.96)
Males Global haplotype association *p*-value: 0.19										
*H1*	A	T	G	A	C	G	0.5715	0.4628	1.00	---
*H2*	A	C	G	A	T	G	0.1015	0.1637	**2.32 (1.10–4.92)**	**0.028** (0.15)
*H3*	G	T	A	A	T	A	0.1541	0.1747	1.52 (0.83–2.77)	0.17 (0.60)
*H4*	A	T	G	A	T	G	0.0990	0.0941	1.33 (0.62–2.84)	0.47 (0.76)
*H5*	G	T	G	A	C	G	0.0000	0.0243	**2.41 (1.28–6.18)**	**<0.0001** (**0.002**)
*H6*	G	T	A	A	C	A	0.0097	0.0202	2.49 (0.31–20.32)	0.39 (0.72)
*H7*	A	T	G	T	T	G	0.0247	0.0252	1.52 (0.39–5.98)	0.55 (0.81)
*rare*	*	*	*	*	*	*	0.0106	0.0020	1.23 (0.25–6.07)	0.80 (0.96)
Females Global haplotype association *p*-value: **0.047**										
*H1*	A	T	G	A	C	G	0.5290	0.4807	1.00	---
*H2*	A	C	G	A	T	G	0.0968	0.1892	**2.13 (1.05–4.30)**	**0.036** (0.15)
*H3*	G	T	A	A	T	A	0.1686	0.1702	0.97 (0.53–1.76)	0.91 (0.96)
*H4*	A	T	G	A	T	G	0.0759	0.0661	0.94 (0.40–2.21)	0.88 (0.96)
*H5*	G	T	G	A	C	G	0.0374	0.0230	0.58 (0.18–1.88)	0.36 (0.72)
*H6*	G	T	A	A	C	A	0.0114	0.0222	2.36 (0.30–18.53)	0.41 (0.72)
*H7*	A	T	G	T	T	G	0.0477	0.0114	**0.20 (0.06–0.66)**	**0.009 (0.05)**
*rare*	*	*	*	*	*	*	0.0000	0.0069	1.01 (0.29–3.55)	0.99 (0.99)

^1^ Rare haplotypes with frequency < 0.01 are not shown and indicated as “*”. ^2^ Odds ratio with 95% confidence intervals adjusted for age, sex, and BMI (codominant genetic model). ^3^ *p*-value (FDR-adjusted *p*-value) adjusted for age, sex, and BMI. Bold is statistically significant *P*- and *Q*-values.

**Table 7:** Genotype frequencies for the studied *RAC1* gene polymorphisms among T2D patients with and without diabetic angiopathy of lower extremities.

*RAC1* SNP ID	Genotype	T2D Patients		*P* (*Q*) ^1^	OR (95% CI) ^2^
		Without DA *n* (%)	With DA *n* (%)		
Entire group					
rs4724800 A>G	A/A	293 (59.4)	552 (57.4)	0.54 (0.94)	1.00
	A/G	173 (35.1)	365 (37.9)		1.13 (0.89–1.43)
	G/G	27 (5.5)	45 (4.7)		0.93 (0.55–1.56)
rs7784465 T>C	T/T	312 (65)	587 (62.7)	0.08 (0.36)	1.00
	T/C	142 (29.6)	316 (33.8)		1.20 (0.93–1.54)
	C/C	26 (5.4)	33 (3.5)		0.65 (0.37–1.13)
rs10951982 G>A	G/G	313 (63.2)	596 (62.3)	0.94 (0.94)	1.00
	G/A	161 (32.5)	321 (33.5)		1.03 (0.81–1.31)
	A/A	21 (4.2)	40 (4.2)		1.09 (0.62–1.94)
rs10238136 A>T	A/A	472 (96.5)	887 (93.8)	**0.02** (0.18)	1.00
	A/T	16 (3.3)	58 (6.1)		**2.16 (1.20–3.87)**
	T/T	1 (0.2)	1 (0.1)		0.45 (0.03–7.19)
rs836478 C>T	C/C	149 (30.6)	271 (28.6)	0.20 (0.60)	1.00
	C/T	243 (49.9)	459 (48.4)		1.03 (0.79–1.34)
	T/T	95 (19.5)	218 (23)		1.31 (0.95–1.81)
rs9374 G>A	G/G	311 (63.5)	592 (62.6)	0.88 (0.94)	1.00
	G/A	159 (32.5)	322 (34)		1.05 (0.82–1.34)
	A/A	20 (4.1)	32 (3.4)		0.93 (0.51–1.70)
Males					
rs4724800 A>G	A/A	123 (60)	157 (57.7)	0.87 (0.94)	1.00
	A/G	72 (35.1)	102 (37.5)		1.09 (0.74–1.62)
	G/G	10 (4.9)	13 (4.8)		1.17 (0.47–2.93)
rs7784465 T>C	T/T	131 (65.5)	160 (60.1)	**0.031** (0.19)	1.00
	T/C	57 (28.5)	104 (39.1)		**1.56 (1.04–2.33)**
	C/C	12 (6)	2 (0.8)		0.16 (0.03–1.02)
rs10951982 G>A	G/G	130 (62.8)	171 (63.6)	0.91 (0.94)	1.00
	G/A	68 (32.9)	86 (32)		0.94 (0.63–1.41)
	A/A	9 (4.3)	12 (4.5)		1.15 (0.44–3.02)
rs10238136 A>T	A/A	192 (94.1)	249 (94)	0.27 (0.69)	1.00
	A/T	11 (5.4)	16 (6)		1.13 (0.50–2.57)
	T/T	1 (0.5)	0 (0)		NA
rs836478 C>T	C/C	56 (27.4)	73 (27)	0.94 (0.94)	1.00
	C/T	104 (51)	136 (50.4)		0.99 (0.64–1.55)
	T/T	44 (21.6)	61 (22.6)		1.08 (0.63–1.86)
rs9374 G>A	G/G	130 (63.1)	172 (63.9)	0.91 (0.94)	1.00
	G/A	68 (33)	86 (32)		0.94 (0.63–1.41)
	A/A	8 (3.9)	11 (4.1)		1.17 (0.42–3.24)
Females					
rs4724800 A>G	A/A	170 (59)	395 (57.2)	0.48 (0.94)	1.00
	A/G	101 (35.1)	263 (38.1)		1.16 (0.86–1.56)
	G/G	17 (5.9)	32 (4.6)		0.83 (0.45–1.56)
rs7784465 T>C	T/T	181 (64.6)	427 (63.7)	0.92 (0.94)	1.00
	T/C	85 (30.4)	212 (31.6)		1.05 (0.77–1.44)
	C/C	14 (5)	31 (4.6)		0.93 (0.48–1.83)
rs10951982 G>A	G/G	183 (63.5)	425 (61.8)	0.86 (0.94)	1.00
	G/A	93 (32.3)	235 (34.2)		1.09 (0.80–1.47)
	A/A	12 (4.2)	28 (4.1)		1.08 (0.53–2.19)
rs10238136 A>T	A/A	280 (98.2)	638 (93.7)	**0.0019** (**0.034**)	1.00
	A/T	5 (1.8)	42 (6.2)		**4.14 (1.60–10.69)**
	T/T	0 (0)	1 (0.2)		NA
rs836478 C>T	C/C	93 (32.9)	198 (29.2)	0.12 (0.43)	1.00
	C/T	139 (49.1)	323 (47.6)		1.04 (0.75–1.44)
	T/T	51 (18)	157 (23.2)		1.48 (0.98–2.23)
rs9374 G>A	G/G	181 (63.7)	420 (62)	0.64 (0.94)	1.00
	G/A	91 (32)	236 (34.9)		1.12 (0.83–1.53)
	A/A	12 (4.2)	21 (3.1)		0.83 (0.40–1.76)

^1^ *p*-value (FDR-adjusted *p*-value) adjusted for age, sex, and BMI. ^2^ Odds ratio with 95% confidence intervals adjusted for age, sex, and BMI (codominant genetic model). Bold is statistically significant *P*- and *Q*-values.

**Table 8:** Estimated common haplotype frequencies of *RAC1* gene in T2D patients with and without diabetic angiopathy of lower extremities.

Haplotype Number	SNPs						T2D Patients		OR (95% CI) ^2^	*P* (*Q*) ^3^
	rs4724800	rs7784465	rs10951982	rs10238136	rs836478	rs9374				
							Without DA	With DA		
							Haplotype Frequency ^1^			
Entire group Global haplotype association *p*-value: 0.53										
*H1*	A	T	G	A	C	G	0.4869	0.4595	1.00	---
*H2*	A	C	G	A	T	G	0.1665	0.1831	1.13 (0.89–1.42)	0.31 (0.67)
*H3*	G	T	A	A	T	A	0.1601	0.1776	1.20 (0.95–1.52)	0.12 (0.67)
*H4*	A	T	G	A	T	G	0.0757	0.0756	1.00 (0.72–1.38)	0.99 (0.99)
*H5*	G	T	G	A	C	G	0.0241	0.0238	1.12 (0.63–1.98)	0.70 (0.86)
*H6*	G	T	A	A	C	A	0.0212	0.0210	0.78 (0.42–1.43)	0.41 (0.67)
*H7*	A	T	G	T	T	G	0.0123	0.0181	1.78 (0.84–3.76)	0.13 (0.67)
*H8*	A	C	G	A	C	G	0.0146	0.0115	0.74 (0.33–1.66)	0.46 (0.67)
*rare*	*	*	*	*	*	*	0.0099	0.0026	0.93 (0.55–1.59)	0.79 (0.86)
Males Global haplotype association *p*-value: 0.83										
*H1*	A	T	G	A	C	G	0.4889	0.4569	1.00	---
*H2*	A	C	G	A	T	G	0.1775	0.1808	1.05 (0.71–1.53)	0.82 (0.86)
*H3*	G	T	A	A	T	A	0.1736	0.1908	1.16 (0.80–1.70)	0.44 (0.67)
*H4*	A	T	G	A	T	G	0.0634	0.0810	1.28 (0.75–2.20)	0.37 (0.67)
*H5*	G	T	G	A	C	G	0.0202	0.0260	1.50 (0.58–3.87)	0.40 (0.67)
*H6*	A	T	G	T	T	G	0.0209	0.0158	0.67 (0.24–1.82)	0.43 (0.67)
*rare*	*	*	*	*	*	*	0.0058	0.0121	0.88 (0.46–1.68)	0.70 (0.86)
Females Global haplotype association *p*-value: 0.078										
*H1*	A	T	G	A	C	G	0.4904	0.4615	1.00	---
*H2*	A	C	G	A	T	G	0.1576	0.1838	1.15 (0.86–1.53)	0.36 (0.67)
*H3*	G	T	A	A	T	A	0.1473	0.1726	1.27 (0.94–1.72)	0.12 (0.67)
*H4*	A	T	G	A	T	G	0.0834	0.0731	0.83 (0.55–1.25)	0.38 (0.67)
*H5*	G	T	G	A	C	G	0.0285	0.0220	0.83 (0.42–1.63)	0.58 (0.80)
*H6*	G	T	A	A	C	A	0.0301	0.0251	0.63 (0.31–1.27)	0.2 (0.67)
*H7*	A	T	G	T	T	G	0.0057	0.0186	**4.63 (1.27–16.84)**	**0.02** (0.44)
*H8*	A	C	G	A	C	G	0.0186	0.0115	0.65 (0.24–1.71)	0.38 (0.67)
*rare*	*	*	*	*	*	*	0.0193	0.0034	1.11 (0.56–2.21)	0.76 (0.86)

^1^ Rare haplotypes with frequency < 0.01 are not shown. ^2^ Odds ratio with 95% confidence intervals adjusted for age, sex, and BMI (codominant genetic model). ^3^ *p*-value (FDR-adjusted *p*-value) adjusted for age, sex, and BMI. Bold is statistically significant *P*- and *Q*-values.

**Table 9:** Estimated common haplotype frequencies of *RAC1* gene in T2D patients with and without diabetic foot syndrome.

Haplotype Number	SNPs						T2D Patients		OR (95% CI) ^2^	*P* (*Q*) ^3^
	rs4724800	rs7784465	rs10951982	rs10238136	rs836478	rs9374				
							Without DFS	With DFS		
							Haplotype Frequency ^1^			
Entire group Global haplotype association *p*-value: 0.12										
*H1*	A	T	G	A	C	G	0.4642	0.4798	1.00	---
*H2*	A	C	G	A	T	G	0.1754	0.2227	1.23 (0.85–1.78)	0.26 (0.90)
*H3*	G	T	A	A	T	A	0.1739	0.1467	0.82 (0.54–1.25)	0.36 (0.90)
*H4*	A	T	G	A	T	G	0.0781	0.0477	0.56 (0.28–1.12)	0.10 (0.55)
*H5*	G	T	G	A	C	G	0.0239	0.0188	0.70 (0.24–2.08)	0.52 (0.94)
*H6*	G	T	A	A	C	A	0.0205	0.0286	1.25 (0.47–3.35)	0.65 (0.94)
*H7*	A	T	G	T	T	G	0.0153	0.0341	2.07 (0.91–4.70)	0.083 (0.55)
*H8*	A	C	G	A	C	G	0.0141	NA	NA	NA
*rare*	*	*	*	*	*	*	0.0055	0.0022	0.67 (0.23–1.91)	0.45 (0.90)
Males Global haplotype association *p*-value: 0.22										
*H1*	A	T	G	A	C	G	0.4677	0.4379	1.00	---
*H2*	A	C	G	A	T	G	0.1819	0.1999	1.19 (0.58–2.43)	0.64 (0.94)
*H3*	G	T	A	A	T	A	0.1845	0.1585	1.01 (0.48–2.14)	0.98 (1.0)
*H4*	A	T	G	A	T	G	0.0769	0.0750	1.01 (0.36–2.83)	0.98 (1.0)
*H5*	G	T	G	A	C	G	0.0252	NA	NA	NA
*H6*	A	T	G	T	T	G	0.0162	0.0632	**4.07 (1.20–13.82)**	**0.025** (0.55)
*rare*	*	*	*	*	*	*	0.0083	0.0000	1.56 (0.52–4.67)	0.42 (0.90)
Females Global haplotype association *p*-value: 0.099										
*H1*	A	T	G	A	C	G	0.4640	0.4986	1.00	---
*H2*	A	C	G	A	T	G	0.1725	0.2325	1.24 (0.81–1.90)	0.33 (0.90)
*H3*	G	T	A	A	T	A	0.1688	0.1410	0.80 (0.48–1.35)	0.40 (0.90)
*H4*	A	T	G	A	T	G	0.0781	0.0360	0.40 (0.16–1.03)	0.06 (0.55)
*H5*	G	T	G	A	C	G	0.0235	0.0274	1.08 (0.37–3.15)	0.89 (1.0)
*H6*	G	T	A	A	C	A	0.0261	0.0371	1.24 (0.44–3.51)	0.68 (0.94)
*H7*	A	T	G	T	T	G	0.0149	0.0202	1.36 (0.41–4.54)	0.61 (0.94)
*H8*	A	C	G	A	C	G	0.0159	NA	NA	NA
*rare*	*	*	*	*	*	*	0.0078	0.0000	0.22 (0.03–1.63)	0.14 (0.62)

^1^ Rare haplotypes with frequency < 0.01 are not shown and indicated as “*”. ^2^ Odds ratio with 95% confidence intervals adjusted for age, sex, and BMI (codominant genetic model). ^3^ *p*-value (FDR-adjusted *p*-value) adjusted for age, sex, and BMI. Bold is statistically significant *P*- and *Q*-values.

## Data Availability

Data supporting reported results are available upon request.

## Supplementary Materials

The following supporting information can be downloaded at: https://www.mdpi.com/article/10.3390/biomedicines11030981/s1, Table S1: Estimated common haplotype frequencies of *RAC1* gene in T2D patients with and without DNF; Table S2: Genotype frequencies for the studied *RAC1* gene polymorphisms among T2D patients with and without diabetic foot syndrome (DFS); Table S3: Associations of *RAC1* haplotypes with plasma redox homeostasis parameters.
