# Supplementary material for: Single Nucleotide Polymorphisms of the RAC1 Gene as Novel Susceptibility Markers for Neuropathy and Microvascular Complications in Type 2 Diabetes
Source: Biomedicines. 2023 Mar 22;11(3):981. doi: 10.3390/biomedicines11030981 (PMC10046239; doi:10.3390/biomedicines11030981)
Supplement: Supplementary file 1 [file biomedicines-11-00981-s001.zip › Supplementary table 2.pdf]

Table S2. Genotype frequencies for the studied *RAC1* gene polymorphisms among T2D patients with and without diabetic foot syndrome (DFS)

RAC1 **SNP ID**	**Genotype**	**T2D patients**		***P*[1]**	**OR (95% CI)[2]**
		**Without DFS n (%)**	**With DFS n (%)**		
Entire group					
rs4724800 A>G	A/A	745 (57.5)	68 (63.5)	0.35 (0.73)	1.00
	A/G	485 (37.5)	36 (33.6)		0.82 (0.54-1.25)
	G/G	65 (5)	3 (2.8)		0.51 (0.16-1.67)
rs7784465 T>C	T/T	801 (63.6)	63 (60)	0.46 (0.73)	1.00
	T/C	406 (32.2)	35 (33.3)		1.09 (0.71-1.68)
	C/C	52 (4.1)	7 (6.7)		1.73 (0.75-3.99)
rs10951982 G>A	G/G	805 (62.3)	69 (65.1)	0.42 (0.73)	1.00
	G/A	433 (33.5)	35 (33)		0.95 (0.62-1.45)
	A/A	55 (4.2)	2 (1.9)		0.43 (0.10-1.79)
rs10238136 A>T	A/A	1210 (94.8)	97 (91.5)	0.17 (0.73)	1.00
	A/T	66 (5.2)	8 (7.5)		1.56 (0.73-3.36)
	T/T	1 (0.1)	1 (0.9)		11.37 (0.70-184.13)
rs836478 C>T	C/C	365 (28.6)	34 (32.1)	0.32 (0.73)	1.00
	C/T	633 (49.6)	45 (42.5)		0.75 (0.47-1.20)
	T/T	278 (21.8)	27 (25.5)		1.04 (0.61-1.77)
rs9374 G>A	G/G	798 (62.5)	70 (65.4)	0.57 (0.73)	1.00
	G/A	432 (33.9)	35 (32.7)		0.93 (0.61-1.42)
	A/A	46 (3.6)	2 (1.9)		0.50 (0.12-2.12)
Males					
rs4724800 A>G	A/A	245 (58.3)	23 (67.7)	0.51 (0.73)	1.00
	A/G	156 (37.1)	10 (29.4)		0.66 (0.31-1.44)
	G/G	19 (4.5)	1 (2.9)		0.55 (0.07-4.32)
rs7784465 T>C	T/T	258 (62.6)	18 (56.2)	0.27 (0.73)	1.00
	T/C	140 (34)	14 (43.8)		1.40 (0.68-2.92)
	C/C	14 (3.4)	0 (0)		0.00 (0.00-NA)
rs10951982 G>A	G/G	266 (63.3)	21 (63.6)	0.95 (0.96)	1.00
	G/A	137 (32.6)	11 (33.3)		1.01 (0.47-2.16)
	A/A	17 (4)	1 (3)		0.72 (0.09-5.75)
rs10238136 A>T	A/A	390 (94.4)	28 (84.8)	0.04 (0.72)	1.00
	A/T	23 (5.6)	4 (12.1)		2.42 (0.78-7.55)
	T/T	0 (0)	1 (3)		NA (0.00-NA)
rs836478 C>T	C/C	110 (26.4)	8 (23.5)	0.61 (0.73)	1.00
	C/T	215 (51.6)	16 (47.1)		1.03 (0.43-2.50)
	T/T	92 (22.1)	10 (29.4)		1.54 (0.58-4.07)
rs9374 G>A	G/G	266 (63.6)	22 (64.7)	0.96 (0.96)	1.00
	G/A	137 (32.8)	11 (32.4)		0.96 (0.45-2.05)
	A/A	15 (3.6)	1 (2.9)		0.76 (0.09-6.15)
Females					
rs4724800	A/A	500 (57.1)	45 (61.6)	0.55	1.00

A>G	A/G	329 (37.6)	26 (35.6)	(0.73)	0.90 (0.54-1.49)
	G/G	46 (5.3)	2 (2.7)		0.49 (0.11-2.08)
rs7784465 T>C	T/T	543 (64.1)	45 (61.6)	0.21 (0.73)	1.00
	T/C	266 (31.4)	21 (28.8)		0.93 (0.54-1.60)
	C/C	38 (4.5)	7 (9.6)		2.22 (0.94-5.28)
rs10951982 G>A	G/G	539 (61.7)	48 (65.8)	0.37 (0.73)	1.00
	G/A	296 (33.9)	24 (32.9)		0.92 (0.55-1.54)
	A/A	38 (4.3)	1 (1.4)		0.30 (0.04-2.26)
rs10238136 A>T	A/A	820 (94.9)	69 (94.5)	0.87 (0.96)	1.00
	A/T	43 (5)	4 (5.5)		1.17 (0.41-3.38)
	T/T	1 (0.1)	0 (0)		0.00 (0.00-NA)
rs836478 C>T	C/C	255 (29.7)	26 (36.1)	0.31 (0.73)	1.00
	C/T	418 (48.7)	29 (40.3)		0.66 (0.38-1.15)
	T/T	186 (21.6)	17 (23.6)		0.90 (0.48-1.72)
rs9374 G>A	G/G	532 (62)	48 (65.8)	0.53 (0.73)	1.00
	G/A	295 (34.4)	24 (32.9)		0.92 (0.55-1.53)
	A/A	31 (3.6)	1 (1.4)		0.37 (0.05-2.81)

[1]Significance level adjusted for age, sex and BMI.

[2]Odds ratio with 95% confidence intervals adjusted for age, sex and BMI (codominant genetic model).

Bold is statistically significant *P*- and *Q*-values.
