# Supplementary material for: Single Nucleotide Polymorphisms of the RAC1 Gene as Novel Susceptibility Markers for Neuropathy and Microvascular Complications in Type 2 Diabetes
Source: Biomedicines. 2023 Mar 22;11(3):981. doi: 10.3390/biomedicines11030981 (PMC10046239; doi:10.3390/biomedicines11030981)
Supplement: Supplementary file 1 [file biomedicines-11-00981-s001.zip › Supplementary table 1.pdf]

Table S1. Estimated common haplotype frequencies of *RAC1* gene in T2D patients with and without DNF

SNPs / *H*	rs4724800	rs7784465	rs10951982	rs10238136	rs836478	rs9374	T2D patients: Without DNF (Haplotype frequency[1])	T2D patients: With DNF (Haplotype frequency[1])	OR (95% CI)[2]	*P*[3] (Q)
							Entire group			
							Global haplotype association p-value: 0.73			
H1	A	T	G	A	C	G	0.4824	0.4744	1.00	---
H2	A	C	G	A	T	G	0.1697	0.1852	1.09 (0.86 - 1.37)	0.48 (0.79)
H3	G	T	A	A	T	A	0.1654	0.1816	1.17 (0.92 - 1.47)	0.20 (0.73)
H4	A	T	G	A	T	G	0.0800	0.0664	0.93 (0.66 - 1.32)	0.70 (1.0)
H5	G	T	G	A	C	G	0.0217	0.0256	1.30 (0.75 - 2.27)	0.35 (0.79)
H6	G	T	A	A	C	A	0.0247	0.0167	0.70 (0.36 - 1.36)	0.29 (0.79)
H7	A	T	G	T	T	G	0.0184	0.0156	1.01 (0.51 - 1.98)	0.98 (1.0)
rare	*	*	*	*	*	*	0.0106	0.0047	0.94 (0.57 - 1.57)	0.83 (1.0)
							Males			
							Global haplotype association p-value: 0.21			
H1	A	T	G	A	C	G	0.4838	0.4312	1.00	---
H2	A	C	G	A	T	G	0.1487	0.1920	1.53 (0.95 - 2.46)	0.078 (0.73)
H3	G	T	A	A	T	A	0.1736	0.1808	1.17 (0.76 - 1.83)	0.48 (0.79)
H4	A	T	G	A	T	G	0.0836	0.1143	1.59 (0.91 - 2.76)	0.10 (0.73)
H5	G	T	G	A	C	G	0.0240	0.0068	0.59 (0.13 - 2.68)	0.50 (0.79)
H6	G	T	A	A	C	A	0.0216	0.0221	1.07 (0.34 - 3.40)	0.91 (1.0)
H7	A	T	G	T	T	G	0.0238	0.0297	1.38 (0.54 - 3.53)	0.50 (0.79)
H8	A	C	G	A	C	G	0.0154	NA	0.00 (-Inf - Inf)	1 (1.0)
rare	*	*	*	*	*	*	0.0049	0	0.85 (0.24 - 3.04)	0.8 (1.0)
							Females			
							Global haplotype association p-value: 0.25			
H1	A	T	G	A	C	G	0.4811	0.4858	1.00	---
H2	A	C	G	A	T	G	0.1854	0.1837	0.98 (0.75 - 1.27)	0.88 (1.0)
H3	G	T	A	A	T	A	0.1591	0.1819	1.14 (0.87 - 1.51)	0.34 (0.79)
H4	A	T	G	A	T	G	0.0777	0.0547	0.71 (0.46 - 1.08)	0.11 (0.73)
H5	G	T	G	A	C	G	0.0199	0.0285	1.54 (0.79 - 2.99)	0.20 (0.73)
H6	G	T	A	A	C	A	0.0272	0.0152	0.57 (0.26 - 1.24)	0.15 (0.73)
H7	A	T	G	T	T	G	0.0147	0.0122	0.72 (0.30 - 1.73)	0.46 (0.79)
rare	*	*	*	*	*	*	0.0072	0.0057	1.08 (0.61 - 1.92)	0.79 (1.0)

[1]Rare haplotypes with frequency < 0.01 are not shown.

[2]Odds ratio with 95% confidence intervals adjusted for age, sex and BMI (codominant genetic model).

[3]Significance level adjusted for age, sex and BMI.

Bold is statistically significant *P*- and *Q*-values. H - haplotype
